# Possible living fossil in Bolivia: A new genus of flea beetles with modified hind legs (Coleoptera, Chrysomelidae, Galerucinae, Alticini)

**DOI:** 10.3897/zookeys.592.8180

**Published:** 2016-05-25

**Authors:** Alexander S. Konstantinov

**Affiliations:** 1 Systematic Entomology Laboratory, USDA, ARS, c/o Smithsonian Institution, National Museum of Natural History, MRC 168, Washington DC, USA

**Keywords:** Flea beetles, fossil beetles, leaping, structure and function of hind leg, Neotropics

## Abstract

A new genus (*Chanealtica*) with three new species (*Chanealtica cuevas*, *Chanealtica ellimon*, and *Chanealtica maxi*) from Bolivia is described and illustrated. It is compared with *Aphthonoides* Jacoby, 1885, *Argopistes* Motschulsky, 1860, *Metroserrapha* Bechyne, 1958, *Psylliodes* Berthold, 1827 and *Psyllototus* Nadein, 2010. Remarkably, based on the available characters, among all the flea beetles, *Chanealtica* is mostly similar to an extinct genus *Psyllototus*. A discussion of diversity and function of the hind leg in flea beetles is provided.

## Introduction

As currently understood, flea beetles (Coleoptera: Chrysomelidae: Galerucinae: Alticini) constitute a polyphyletic group (about 9900 valid species assigned to 577 valid genera) most members of which are characterized by an enlarged metafemora with metafemoral spring inside and an ability to leap (Konstantinov 1994). In beetles and other small insects the leaping ability is understood as high-speed escape reflex (Brackenbury and Wang 1995). It is reasonable to assume that enlarged metafemora with metafemoral spring and associated leaping ability, at least in part, are responsible for extraordinary flea beetle radiation (they are the most species rich family level taxon among about 36,000 named leaf beetle species). The associated diversification resulted in a remarkable variety of forms and shapes of the flea beetle bodies, but particularly in the structure of the hind legs. Flea beetle hind legs differ not only in proportions of metafemora, tibiae and tarsi, but also in shapes, armaments and sites of attachment of hind leg structures, particularly that of the metatarsi.

The current representatives of the vast majority of flea beetle genera have their first metatarsomere attached to the apex of the metatibia (just like in much of all beetles). In Monoplatina flea beetles (47 valid genera and 583 species), the first metatarsomere is attached slightly away from the metatibial apex. Until now, leaf beetles from only four extant genera (*Aphthonoides* Jacoby, 1885, *Argopistes* Motschulsky, 1860, *Metroserrapha* Bechyne, 1958, and *Psylliodes* Berthold, 1827) have their first metatarsomere attached to the metatibia at a significant distance from its apex.

*Chanealtica*, a new genus discovered in Bolivia (described in this paper) possesses the same character state. The overall structure of the hind leg in *Chanealtica* is mostly similar to that of *Psylliodes*. However all 204 recent *Psylliodes* species have 10 antennomeres in their antennae, a condition rarely observed in flea beetles (e.g. *Deciplatus* Linzmeier & Konstantinov, 2009 and *Monotalla* Bechyne, Konstantinov et al. 2015), while *Chanealtica* has 11.

Remarkably, based on the characters that are available for observation, among all the flea beetles, the only one with combination of 11 antennomeres and the first metatarsomere attached to the metatibia at a significant distance from its apex is a recently described from Baltic amber, extinct flea beetle genus *Psyllototus* Nadein (in Nadein and Perkovsky 2010). Although, details of the *Psyllototus* head and ventral side of the body are not available for study. In total, 14 species in 11 genera of fossil flea beetle are known so far (Bukejs and Konstantinov 2013, Bukejs et al. 2015). Morphologically they are close to most typical flea beetles, e.g. *Altica* Geoffroy, with antebasal transverse impression on pronotum and the first metatarsomere attached to the apex of the metatibia. Overall *Psyllototus* is as different from fossil flea beetles as *Chanealtica* from recent.

## Material and methods

Dissecting techniques, measurements, and terminology follow Konstantinov (1998). Observations were made with a Zeiss Discovery V20 microscope and digital images were taken with an AxioCam HRC digital camera attached to it. Habitus illustration was produced after a technique described in Litwak and Harel (2013). Specimens are deposited in the Museo de Historia Natural Noel Kempff Mercado, Universidad Autonoma “Gabriel Rene Moreno”, Santa Cruz, Bolivia (MNKB) and National Museum of Natural History, Smithsonian Institution, Washington DC, USA (USNM).

## Results

### Chanealtica gen. n.

Taxon classificationAnimaliaColeopteraChrysomelidae

http://zoobank.org/40F47B5F-3D6F-4711-8D1F-F98681353855

Figs 1–5 , 6–10 , 11–12 , 13–18 , 19–25 , 26 , 27–31 , 32–36

#### Description.

Body length: 2.59–3.29 mm; body width (widest point of elytra): 1.35–1.67 mm. Pronotum width to length ratio: 1.66–1.77. Width of elytra at base (in middle of humeral calli) to width of pronotum at base ratio: 1.20–1.21.

Body light ochre with last eight antennomeres, elytral apices (in *Chanealtica cuevas*) and bases of metatibia dark brown.

Head with midcranial and frontal sutures absent. Supraorbital sulcus deep. Orbital sulcus visible, situated close to eye. Supracallinal sulcus absent. Supraantennal sulcus shallow, poorly developed. Midfrontal sulcus developed only dorsally, absent ventrally, antennal calli completely separated only dorsally, connected ventrally. Suprafrontal sulcus poorly developed. Frontolateral sulcus well developed. Antennal callus long, oblique, nearly triangular, entering interantennal space. Surface of antennal callus covered with fine, long, transverse wrinkles, situated slightly above surface of vertex. Vertex densely and evenly covered with deep, but poorly delineated punctures. Frontal ridge and vertex separated by antennal calli. Width of frontal ridge to width of antennal socket (including surrounding ridges) ratio 0.85 - 0.88. Frontal ridge short, in lateral view almost straight. Area below antennal socket concave. Orbit normally wide, nearly as wide as transverse diameter of antennal socket. Distance between eyes above antennal sockets to transverse diameter of eye in frontal view ratio 3.25 - 3.32. Sides of head below eyes converging ventrally. Labrum flat, with 2 pairs of long setae; anterior margin complete, with slight indentation on upper surface. Apical maxillary palpomere conical. Preapical maxillary palpomere wider than apical palpomere. Antennal sockets situated below middle of eye. Antenna filiform, with 11 antennomeres. Length of antenna over pronotum reaching beyond middle of elytron.

Pronotum wider than long, with sides slightly convex to nearly straight. Pronotal base slanted from posterolateral callosities, straight in middle. Lateral margin narrowly explanate, without setae. Anterolateral callosity relatively short, nearly perpendicular to lateral margin. Posterolateral callosity short, not protruding beyond lateral margin. Pronotal punctures relatively dense, shallow. Procoxal cavities open. Intercoxal prosternal process short, narrowing posteriorly, does not extend beyond procoxae, lateral sides straight, posterior end rounded.

Scutellum present. Elytron with punctation confused and few irregular poorly defined longitudinal ridges. Elytra at base wider than base of pronotum. Humeral calli well developed. Basal calli present, poorly separated from elytral disc. Epipleura slightly oblique outwardly, gradually narrowing from base to apex, reaching end of elytron side, but not apex. Mesosternum without elevated projection in middle, flat, in shape similar to intercoxal prosternal process. Metasternum anteriorly without elevated projection in middle and not projecting forward hiding mesosternum.

First adominal ventrite free. Abdominal ventrites about equally long. First abdominal ventrite between coxae without longitudinal ridges, with apex truncate. Last visible tergite without longitudinal groove in middle. Male last abdominal sternite with transverse ridge. In female ridge absent, instead last abdominal sternite forms posteriorly directed lobe with slightly converging sides.

Pro- and mesotibiae canaliculate dorsally. Protibial and mesotibial spurs absent. Metatibia strongly curved. Metatibia in cross section around its middle more or less triangular. Dorsal surface flat to concave. Bristles present on lateral and mesal sides of metatibiae. Metatarsomere 1 attached away from metatibial apex, distance between metatarsal attachment and metatibial apex about 0.3 of metatibial length. Apical spur of metatibia simple, wide, ending in one tooth, situated in middle, but directed medially. Metatarsomere 1 nearly round, longer than rest of metarsomeres together. Claws appendiculate.

Median lobe of aedeagus in cross section somewhat flat, with shallow impressions near apex.

Spermatheca with distinct border between receptacle and pump. Receptacle slightly longer than wide, much wider but about as long as pump, ovoid. Spermathecal duct very wide at base, curved, without coils. Vaginal palpi not fused medially, gradually narrowing posteriorly with a few bristles at apex. Tignum with narrow and relatively short base and wide and long sclerotization posterior.

#### Etymology.

I named the genus after the Chané, a native ethnic group of people, whose traditional lands are in the plains and valleys between the Gran Chaco and the Andes in Bolivia and also in northern Argentina and Paraguay. The name is feminine.

#### Type species.

*Chanealtica cuevas* Konstantinov, sp. n.

#### Host plant.

*Tecoma stans* (L.) Juss. (Bignoniaceae) (Figs 11, 12).

#### Remarks.

*Chanealtica* is markedly different from most known flea beetle genera. However, based on the general structure of the hind tibia and tarsi, *Chanealtica* is similar to extant *Aphthonoides*, *Argopistes*, *Metroserrapha*, *Psylliodes*, and extinct *Psyllototus*. Representatives of all these genera have their metatarsi attached not at the apex of the metatibia but before the apex (Figs 37–42). Many flea beetles of the subtribe Monoplatina have their metatarsi attached to the metatibia before its apex, but the distance from the place of the attachment to the apex is very short in Monoplatina compared to that of *Aphthonoides*, *Argopistes*, *Chanealtica*, *Metroserrapha*, *Psylliodes*, and *Psyllototus*. In addition Monoplatina are clearly different based on a number of phylogenetically important characters (such as globose fourth meso- and metatarsomere, densely setose dorsal surface of the body etc.).

*Chanealtica* can be easily separated from *Aphthonoides* based on a much larger size, metatibia much longer than metatibial spur, confused elytral punctures and numerous features of the head and thorax (*Aphthonoides* has much smaller body, metatibia much shorter than metatibial spur, elytral punctures arranged into striae). *Chanealtica* clearly differs from *Argopistes* in having an elongate and flat body in lateral view, prognathous head, short frontal ridge, narrow and oblique outwardly elytral epipleura (in *Argopistes* body round and convex in lateral view, head opistognathous, frontal ridge long, elytral epipleura wide and oblique inwardly).

In the site of the metatarsal attachment and general shape, metatibia of *Chanealtica* is more similar to that of *Metroserrapha* and *Psylliodes*. In these genera, the metatarsi are attached at about basal 2/3 of the metatibia; the dorsal surface of the metatibia before the metatarsal attachment is flat or canaliculate with lateral margins lacking denticles; the dorsal surface posterior to the metatarsus is deeply channeled and lateral margins are covered with denticles or a row of stiff and short bristles; the metatibial apex is armed with a large, acute spur. *Chanealtica* can be easily distinguished from *Metroserrapha* and *Psylliodes* based on a large body flat in lateral view, confused elytral punctuation, short frontal ridge, anterolateral callosity relatively short, nearly perpendicular to lateral margin of pronotum. In addition, *Chanealtica* and *Metroserrapha* species have 11 antennomeres while all *Psylliodes* species have 10.

The last abdominal sternite contains the unique feature of *Chanealtica*. In male the apex of the sternite is swollen into a transverse ridge. In female the ridge is absent, instead the last abdominal sternite forms a posteriorly directed lobe, which is common in males of many other flea beetle genera (e.g. *Longitarsus* Berthold).

Based on the characters that are available for observation in amber preserved specimens, among all the flea beetles, *Chanealtica* is mostly similar to an extinct genus *Psyllototus*. They share two most vivid character states: structure of the hind leg and antennae with 11 antennomeres. Details of *Psyllototus* head and ventral side of the body are not available for study.

### Chanealtica cuevas sp. n.

Taxon classificationAnimaliaColeopteraChrysomelidae

http://zoobank.org/421BC2E0-630A-4C73-A0FC-73E5DA33C2AF

Figs 1–5 , 6–10 , 11–12

#### Description.

Body length 2.75–3.24 mm. Width 1.35–1.67 mm. Color light ochre with last eight antennomeres, elytral apices and bases of metatibia dark brown. Metatibial apices black.

Proportions of male antennomeres 1–6 as follows: 13:6:7:11:13:13.

Pronotum with lateral margins slightly and evenly convex, at base almost as wide as at apex. Length to width ratio of first protarsomere of male 1.78.

Median lobe of aedeagus in ventral view relatively narrow, widening relatively abruptly (Fig. 5). Apex in ventral view with low, slightly channeled ridge separating two wide and shallow impressions lateral of it. Apex in lateral view with distinct knob facing ventrally. Spermathecal receptacle with basal part significantly smaller than apical (Fig. 8). Narrow, anterior part of tignum shorter than posterior part (Fig. 9).

**Figures 1–5.**
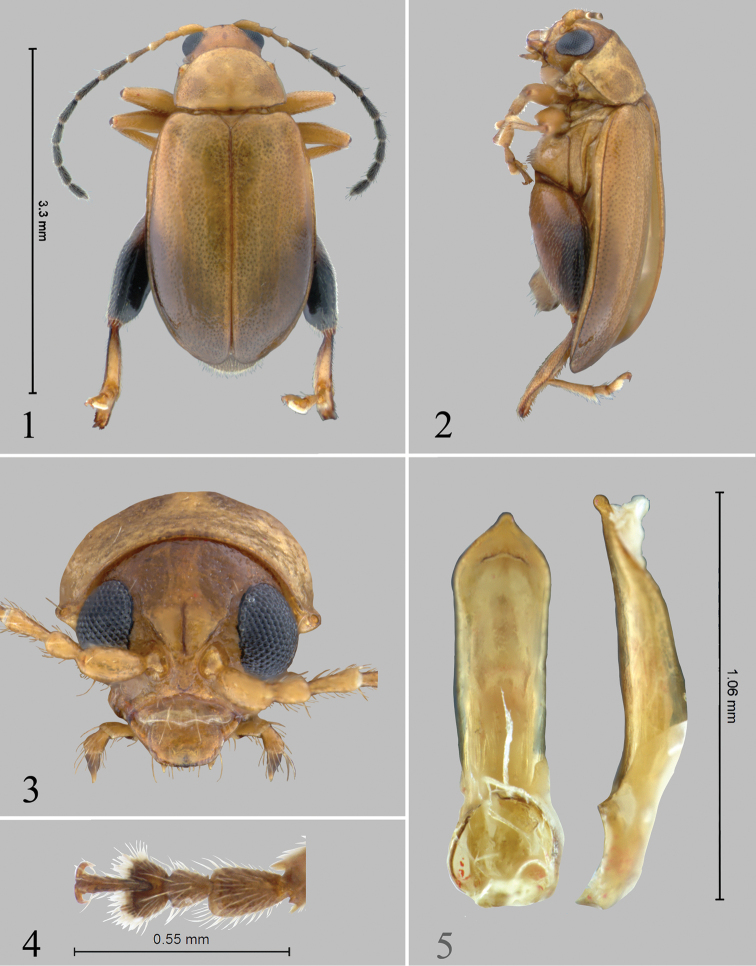
*Chanealtica cuevas*. **1** Habitus dorsal **2** Habitus lateral **3** Head, frontal view **4** Front tarsi, male **5** Aedeagus, ventral and lateral views.

**Figures 6–10.**
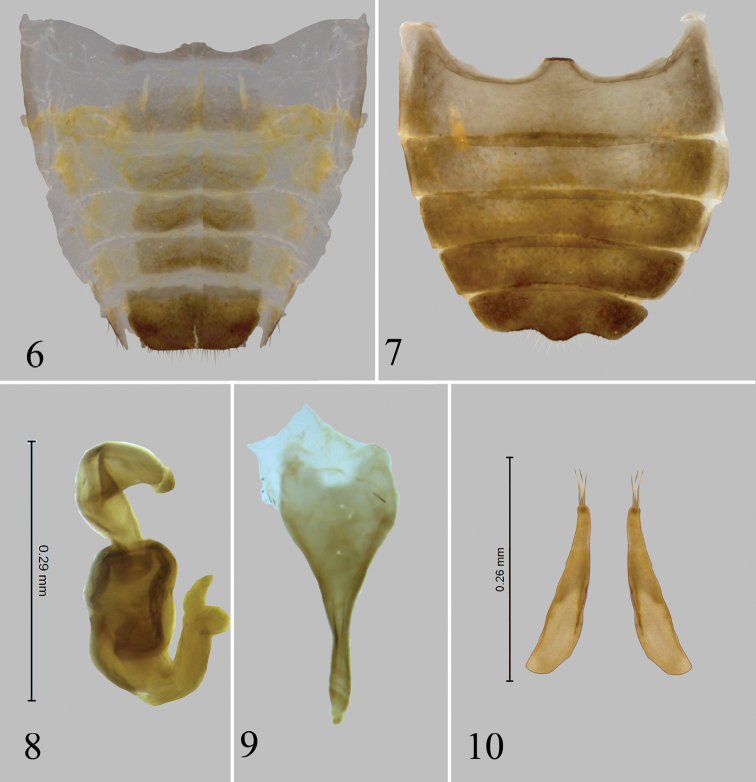
*Chanealtica cuevas*. **6** Abdominal tergites, female **7** Abdominal sternites, female **8** Spermatheca **9** Tignum **10** Vaginal palpi.

#### Host plant.

**Figures 11–12.**
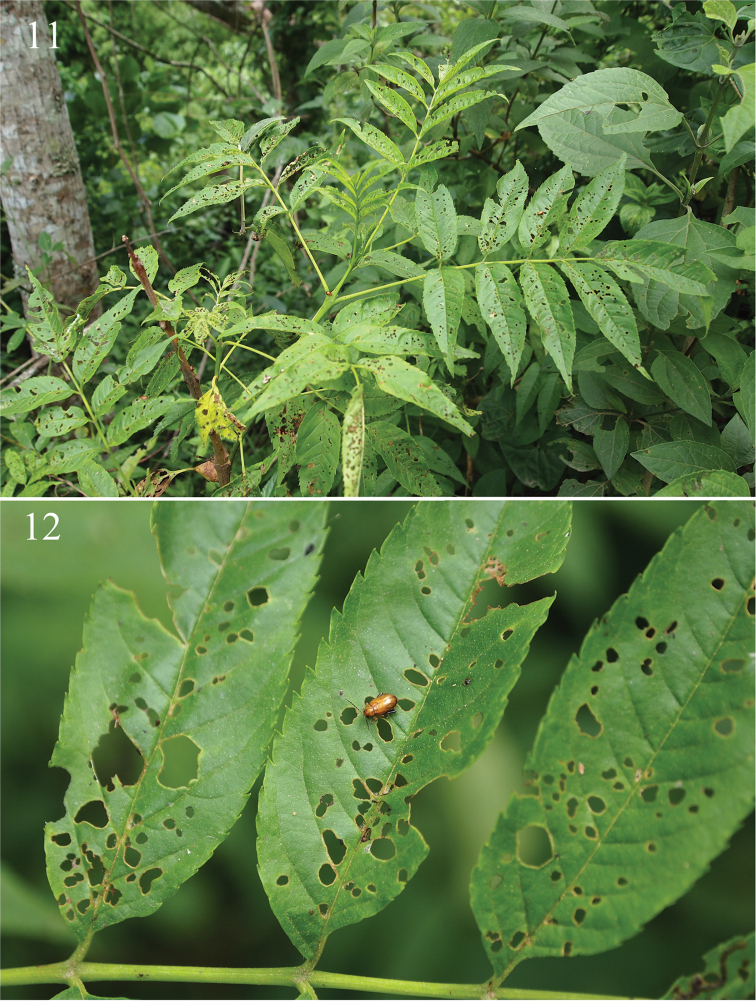
*Chanealtica cuevas*. Host plant, *Tecoma stans* (L.) Juss. (Bignoniaceae).

*Tecoma stans* (L.) Juss. (Bignoniaceae) (Figs 11, 12).

#### Etymology.

This species is named after the type locality.

#### Material examined.

Holotype, male. Labels: 1) BOLIVIA: Santa Cruz Dept. Florida Prov., 7 km SE of Cuevas WP-407, 1332m, 18°12.414'S, 63°40.808'W, 27.XI.2013, leg. A. Konstantinov; 2) Holotype *Chanealtica cuevas* n. sp. des. A. Konstantinov, 2016 (will be deposited at MNKB, currently at USNM). Paratypes 38 specimens. Same labels as holotype (19 USNM, 2 MNKB). Paratypes. Labels: 1) BOLIVIA: Santa Cruz Dept. Florida Prov., 7 km SE of Cuevas WP-408, 1350m, 18°12.734'S, 63°40.776'W, 28.XI.2013, leg. A. Konstantinov; 2) Paratype *Chanealtica cuevas* n. sp. des. A. Konstantinov, 2016 (15 USNM, 2 MNKB).

### Chanealtica ellimon sp. n.

Taxon classificationAnimaliaColeopteraChrysomelidae

http://zoobank.org/AA7F0AC0-8871-4049-9361-FFB101C2E1EB

Figs 13–18 , 19–25

#### Description.

Body length 2.64–3.08 mm. Width 1.51–1.62 mm. Color light ochre with last eight antennomeres and apices of metatibia dark brown.

Proportions of male antennomeres 1–6 as follows: 13:6:7:10:13:13.

Pronotum with lateral margins slightly convex, at base more so than at apex, also narrower at apex than at base. Length to width ratio of first protarsomere of male 1.98.

Median lobe of aedeagus in ventral view relatively narrow, widening relatively gradually (Fig. 19). Apex in ventral view with low, not channeled ridge ending into shallow impression lateral and behind it. Two elongate and shallow impressions situated on sides of apical part of median lobe. Apex in lateral view without distinct sphere, bend ventrally. Spermathecal receptacle with basal part significantly smaller than apical (Fig. 21). Narrow, anterior part of tignum about as long as posterior part (Fig. 24).

**Figures 13–18.**
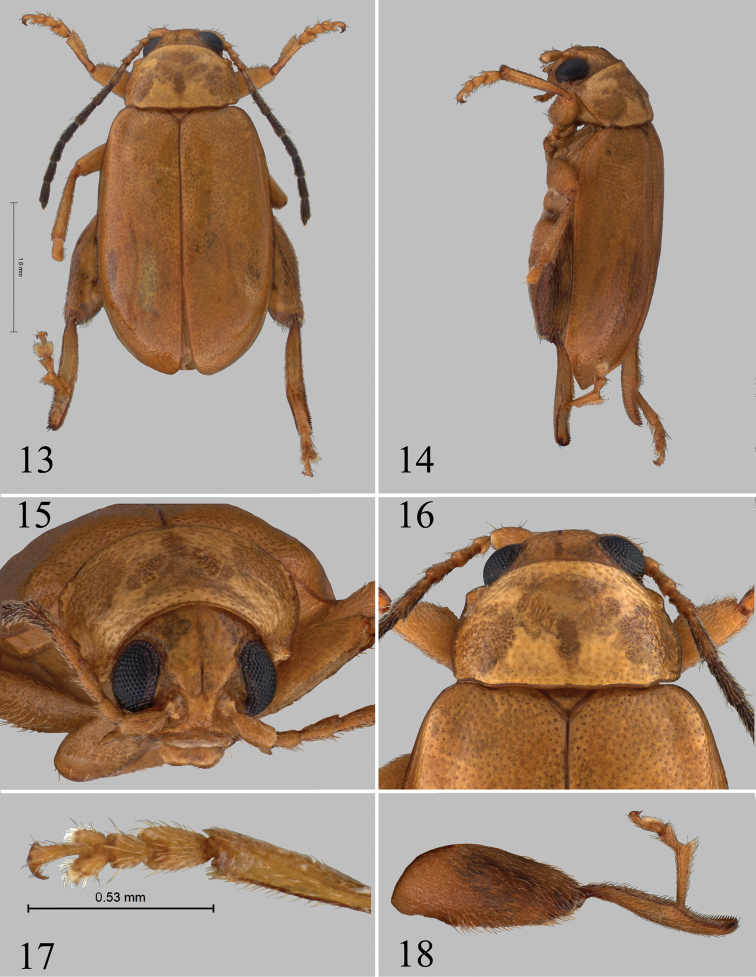
*Chanealtica ellimon*. **13** Habitus dorsal **14** Habitus lateral **15** Head, frontal view **16** Pronotum **17** Front tarsi, male **18** Hind leg, male.

**Figures 19–25.**
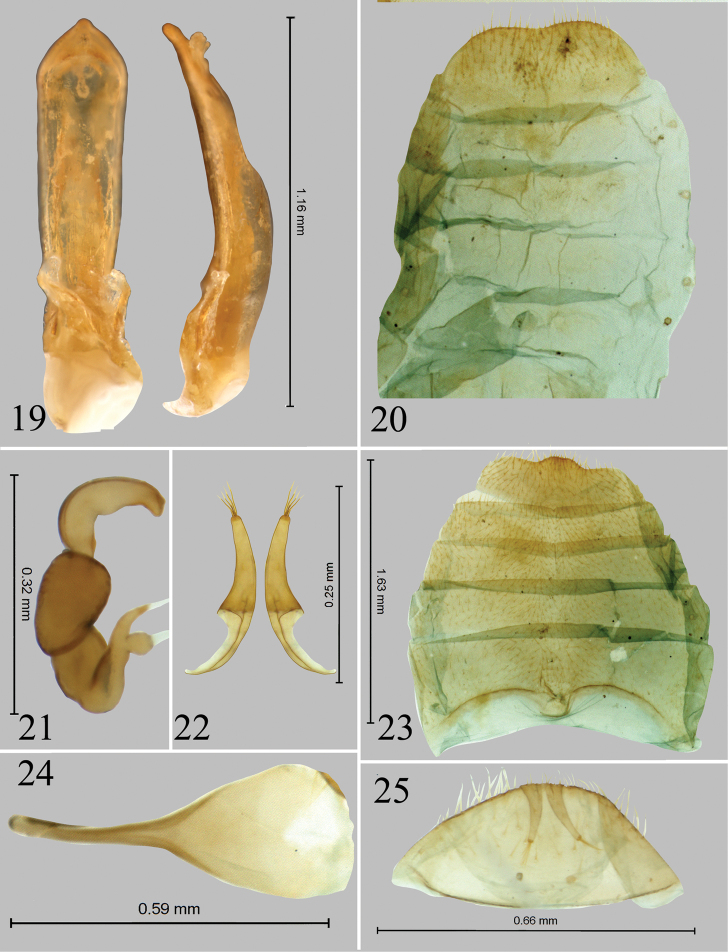
*Chanealtica ellimon*. **19** Aedeagus, ventral and lateral views **20** Abdominal tergites, female **21** Spermatheca **22** Vaginal palpi **23** abdominal sternites, female **24** Tignum **25** 8^th^ abdominal tergite.

#### Etymology.

This species is named after the type locality.

#### Material examined.

Holotype male. Labels: 1) Bolivia: Santa Cruz Dept. Cordillera Prov., Mirador, El Limón 845 meters, 19°04'S, 63°28'W 8.XI.2007, leg. A. Konstantinov; 2) Holotype *Chanealtica ellimon* sp. n. des. A. Konstantinov 2016 (will be deposited at MNKB, currently at USNM). Paratypes with same labels as holotype (3 USNM, 1 MNKB).

### Chanealtica maxi sp. n.

Taxon classificationAnimaliaColeopteraChrysomelidae

http://zoobank.org/856EF8FF-617D-4555-ABB2-9C8098C962A1

Figs 26 , 27–31 , 32–36

#### Description.

Body length 2.59–3.29 mm. Width 1.51–1.72 mm. Color light ochre with last eight antennomeres and apices of metatibia dark brown.

Proportions of male antennomeres 1–6 as follows: 14:6:8:10:14:13.

Pronotum with lateral margins nearly straight, at base wider than at apex. Length to width ratio of first protarsomere of male 1.43.

Median lobe of aedeagus in ventral view relatively wide, widening relatively gradually (Fig. 29). Apex in ventral view without ridge, but with wide and shallow impression. Apex in lateral view with indistinct knob slightly bent ventrally. Spermathecal receptacle with basal part significantly smaller than apical (Fig. 34). Narrow, anterior part of tignum shorter than posterior part (Fig. 35).

**Figure 26.**
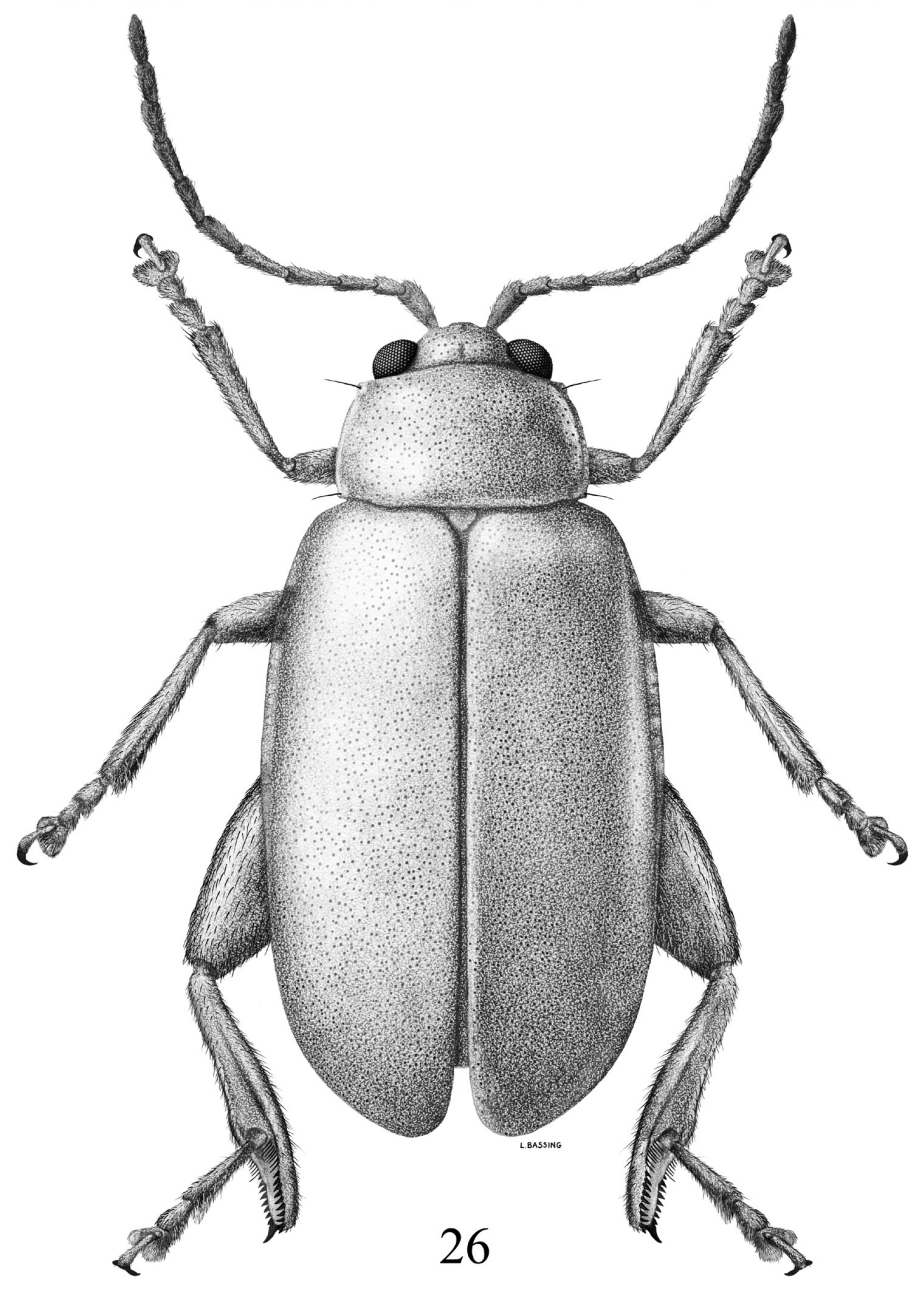
*Chanealtica maxi*. Dorsal habitus.

**Figures 27–31.**
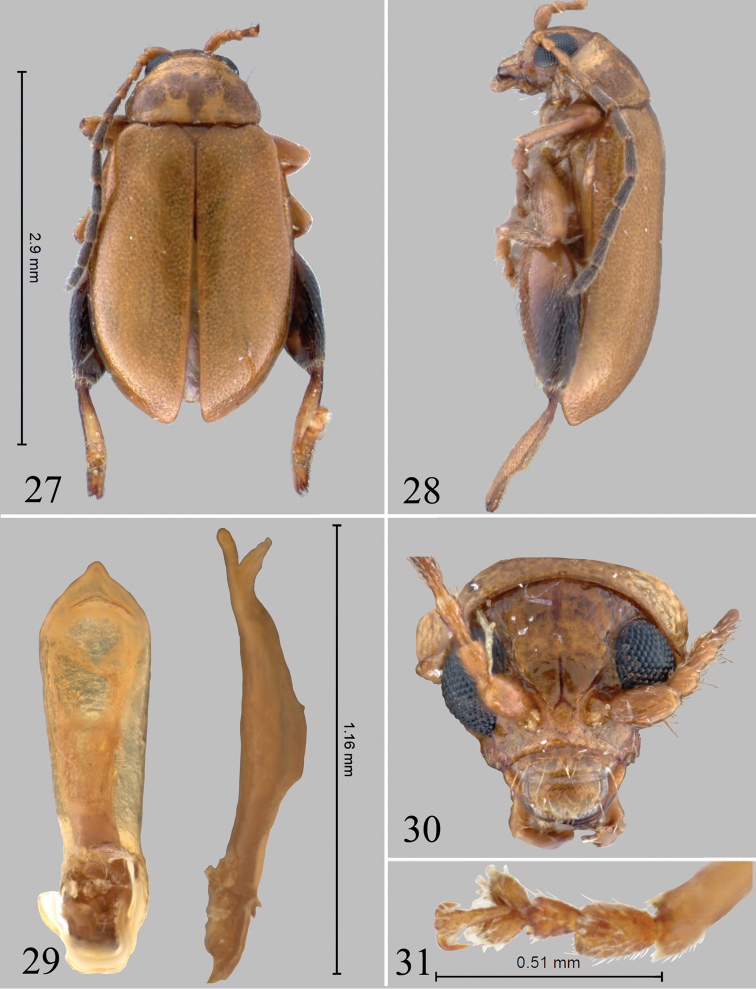
*Chanealtica maxi*. **27** Habitus dorsal **28** Habitus lateral **29** Aedeagus, ventral and lateral views **30** Head, frontal view **31** Front tarsi, male.

**Figures 32–36.**
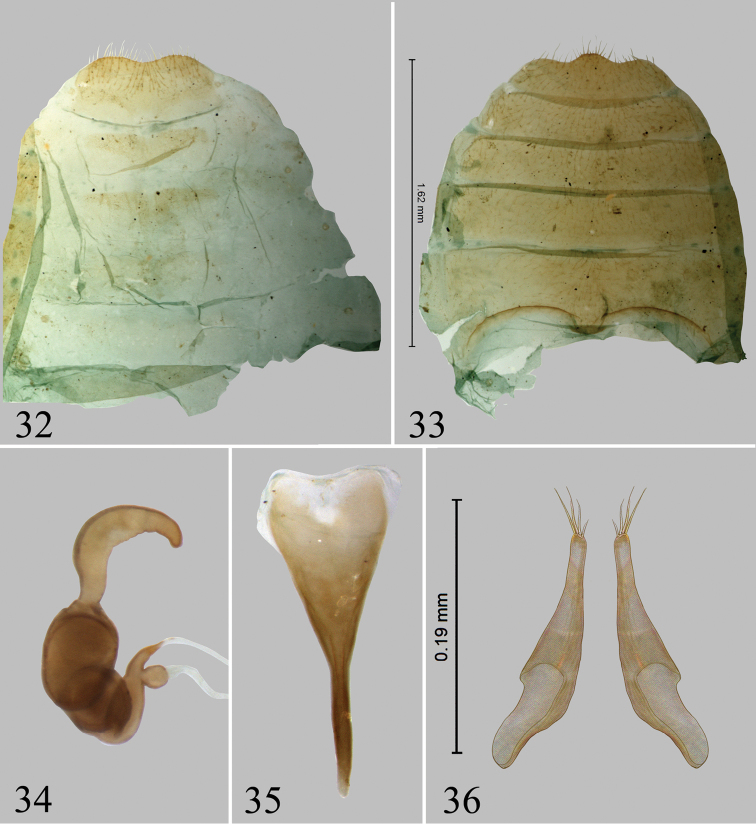
*Chanealtica maxi*. **32** Abdominal tergites, female **33** Abdominal sternites, female **34** Spermatheca **35** Tignum **36** Vaginal palpi.

#### Etymology.

This species is named after Max Joseph Miles, the “newest” member of the Konstantinov/Miles family.

#### Material examined.

Holotype female. Labels: 1) Bolivia: Santa Cruz Dept. Florida Prov., Refugio Los Volcanes, 4 km N of Bermejo 1000–1300 m 29.X.2007 18°06'S, 63°36'W leg. S. Lingafelter & N. Woodley; 2) Holotype *Chanealtica maxi* sp. n. des. A. Konstantinov 2016 (will be deposited at MNKB, currently at USNM). Paratypes: 1) Bolivia: Santa Cruz Dept. Florida Prov., Refugio Los Volcanes, 4 km N of Bermejo, 1000–1300 m 28.X.2007, 18°06'S, 63°36'W leg. A. Konstantinov; 2) Paratype *Chanealtica maxi* sp. n. des. A. Konstantinov 2016 (2 USNM). 1) Bolivia: Santa Cruz Dept. Florida Prov., Refugio Los Volcanes, 4 km N of Bermejo, 1000-1300 m 4.XI.2007, 18°06'S, 63°36'W Laurel trail leg. A. Konstantinov; 2) Paratype *Chanealtica maxi* sp. n. des. A. Konstantinov 2016 (2 USNM).

**Figures 37–42.**
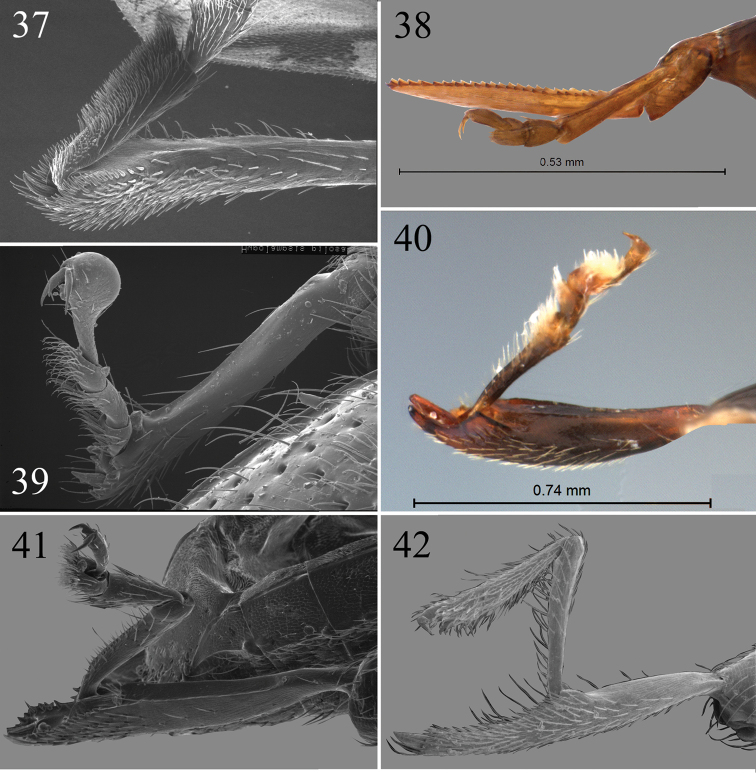
Examples of the metatibiae and metatarsi in flea beetles. **37** *Aphthona nonstriata* (Goeze) **38** *Aphthonoides* sp. **39** *Hypolampsis pilosa* (Illiger) **40** *Argopistes* sp. **41** *Psylliodes luteola* (Muller) **42** *Metroserrapha* sp.

### Key to *Chanealtica* species

**Table:** 

1	Elytral apices dark brown, darker than rest of elytra. Apex of median lobe of aedeagus in ventral view with low, slightly channeled ridge separating two wide and shallow impressions lateral of it. Apex in lateral view with distinct knob facing ventrally	***Chanealtica cuevas***
–	Elytral apices light ochre, as light as rest of elytra. Apex of median lobe of aedeagus in ventral view without low ridge, or if ridge present, its is not chanelled	**2**
2(1)	Pronotum with lateral margins slightly convex, at base more so than at apex. Median lobe of aedeagus in ventral view relatively narrow. Apex of median lobe of aedeagus in ventral view with low, not channeled ridge ending into shallow impression lateral and behind it. Two elongate and shallow impressions situated on sides of apical part of median lobe (Fig. 19)	***Chanealtica ellimon***
–	Pronotum with lateral margins nearly straight, at base wider than at apex. Median lobe of aedeagus in ventral view relatively wide. Apex of median lobe of aedeagus in ventral view without ridge, but with wide and shallow impression (Fig. 29)	***Chanealtica maxi***

## Discussion on flea beetle hind leg diversity and jumping

It seems reasonable to assume that the diversity of the hind legs in flea beetles (Figs 37–42) (exceeding that of many other much more species rich groups of beetles that are not in habit of leaping) is somehow influenced by their leaping. Indeed, the hind leg modifications in beetles are associated with their particular functions (Crowson 1981).

A study of the kinematics of the flea beetle jump (Brackenbury and Wang 1995) suggested that there is a difference in the jump parameters between species with different hind leg structure. Schmitt (2004) argued that three variables affect the flea beetle jumping performance: 1) metafemoral spring structure (a more slender spring with a seemingly solid ventral lobe, as in *Longitarsus* species, seem to result in more efficient jump); 2) muscle volume (the greater it is, the more effective the jump); and 3) length of the hind leg including tarsi (as in *Longitarsus* species with a highly elongate first metatarsomere). This contradicts a bit to an observation by Alexander (1995) who, based on mathematical models, investigated the effect of the muscle properties, leg design and jumping techniques on the jump height in humans, other vertebrates and insects. For animals without catapult jumping mechanism “… longer legs makes a higher jumps possible and additional leg segments, such as the elongated tarsi of bushbabies and frogs, increase jump heights …” (Alexander 1995). However, according to Alexander (1995), for insects with a catapult jumping mechanism (which all flea beetle posses) “the advantage of long legs might be small”.

Schmitt (2004) also pointed out that *Psylliodes* hind leg structure with metatarsus attached away from the metatibial apex is most likely another adaptation for an effective jump. In both Schmitt (2004) and Brackenbury and Wang (1995) studies, *Longitarsus* and *Psylliodes* species exhibit the greatest take-off acceleration, velocity and translational energy (Table 1, Brackenbury and Wang 1995). Brackenbury and Wang (1995) call them “high-speed jumpers” compared to “low-speed jumpers” all of which have a more common position of relatively short metatarsi, right at the apex of the metatibia. According to Brackenbury and Wang (1995) and based on unpublished but available to me recordings of the flea beetle jumps, beetles push themselves from the substratum with the metatibial apex. Metatarsi do not participate in the initial push and sometime are lifted from the substratum. They may function to prevent slippage (Brackenbury and Wang 1995). It may be possible that high-speed jump with tarsi right at the end of the metatibia may not be as efficient or result in tarsal injury, while position of the tarsi away and at a distance from the metatibial apex may allow for a more efficient jump or be safer for a more powerful jump without a possibility of an injury.

A discovery of *Chanealtica*, another flea beetle genus with metatarsi attached away from the metatibial apex, provides a new example of this remarkable jumping leg adaptation.

## Supplementary Material

XML Treatment for Chanealtica

XML Treatment for Chanealtica cuevas

XML Treatment for Chanealtica ellimon

XML Treatment for Chanealtica maxi
